# Novel 3D printing method to reinforce implant‐supported denture fiberglass as material for implant prosthesis: A pilot study

**DOI:** 10.1002/cre2.568

**Published:** 2022-04-19

**Authors:** Andrea Nicali, Giulia Pradal, Gianluca Brandolini, Andrea Mantelli, Marinella Levi

**Affiliations:** ^1^ Dipartimento di Scienze Biomediche, Chirurgiche e Odontoiatriche Università degli Studi di Milano Milano Italy; ^2^ Biart s.n.c. Galbiate (LC) Italy; ^3^ Department of Chemistry, Materials and Chemical Engineering “Giulio Natta” Politecnico di Milano Milano Italy

**Keywords:** 3D‐printed dental structure, dental implants, fiber endostructure, fiberglass prosthesis, fiber prosthetic frameworks

## Abstract

**Objectives:**

Despite a large amount of materials and methods to make an implant‐supported denture, nowadays there is no gold standard. Every solution has pros and cons that guide the clinician and the technician to choose the best solution for a single case. The aim of this study was to evaluate the mechanical characteristics of the fiber‐reinforced composite superstructure made by using a novel three‐dimensional (3D) printing technology able to create a reinforcing structure patient‐specific, more reliable, structurally optimized, and faster than conventional methods.

**Materials and Methods:**

To evaluate mechanical performances of 3D‐printed fiberglass, mechanical characterization of 3D‐printed material was performed. Before proceeding with the realization of the final prosthesis, five specimens were created on which the tensile test and volumetric fiber content measurement were performed. Then denture reinforcement 3D printing process began. Initially, the robot prints layers of fiber. Finally, the obtained 3D‐printed reinforcement structure was finalized in the lab.

**Results:**

The prosthesis obtained through this process was lighter than a traditional prosthesis, there was a greater chemical adhesion between resin and 3D‐printed reinforcement structure and a better result was obtained from an esthetic point of view.

**Conclusions:**

The outcomes we obtained endorse its performance both mechanical and esthetic. The entire process is automatic and does not require human operation thanks to specific software programming.

## 1 INTRODUCTION

The introduction of computer‐aided design/computer‐aided manufacturing led to a more accurate manufacturing of prosthetic frameworks, greater accuracy of dental restorations, and in particular, implant‐supported prosthesis (Douglass et al., 2002).

The medical industry is one of the industries where design freedom and customization can be used. Both of them are advantages of additive manufacturing. Moreover using scans taken from the actual patient's mouth dentists and dental laboratories are able to build accurate and tailored solutions to fix dental problems. Currently, the most widely used solutions are esthetic aligners, crowns, and bridges. By 2027, three‐dimensional (3D) printing technologies will become the leading production source for dental restorations worldwide, surpassing milling and analog fabrication (Smartech Markets Publishing, 2018).

From a technical point of view, a denture, removable or fixed, is composed of different parts: some of them are functional and others aesthetical. In order not to damage or break the prosthesis under loads or fatigue, these parts are built using a framework reinforcing the inner structure. Superstructures of an implant‐supported denture commonly consist of a framework veneered with ceramic or composite resin facing. At present, there are a lot of materials that can be used to manufacture frameworks: zirconium oxide, titanium, polyetheretherketone, polymethylmethacrylate, and chrome‐cobalt.

A novel alternative to metal‐supported and ceramic restorations in implant‐supported fixed partial dentures (FPDs) is fiber‐reinforced composite (FRC) designs (Erkmen et al., 2011; Ruyter et al. 1986) FRC materials, which had been successfully used in a variety of commercial applications, as in aerospace applications, have been widely used also in prosthetic and restorative dentistry. Glass FRC has been suggested even for implant‐supported fixed prostheses (Erkmen et al., 2011; Freilich et al., 2002; van de Werken et al., 2020). FRC prostheses have been presented with a framework composed of fiber bundles preimpregnated with a resin matrix and a veneering composite that embeds the FRC framework (Freilich, 2000). Laboratory studies have shown that FRC materials exhibit flexure strength that is greater than or comparable to metal alloys (Erkmen et al., 2011; Fontijn‐Tekamp et al., 2000) evaluated that the fracture strength of glass FRC FPD on dental implants was almost three times higher than the maximum chewing force measured in young patients with natural dentitions (400N) (Fontijn‐Tekamp et al., 2000). The use of fiber composite technology for FPDs is an alternative to metal–alloy, metal–ceramic, or all‐ceramic restorations (Fischer et al., 2004). Moreover, FRC has been suggested to absorb energy from the masticatory cycle due to the lower flexural modulus of the material (Meric et al., 2005). Composite veneer materials have distinct advantages over porcelain veneers; the former being less brittle, do not wear the opposing dentition, and chemically bond to the FRC framework (Freilich et al., 1998).

Recently FRC was found to have better stress distribution than other materials, such as glass–ceramic, gold, alumina, and zirconia (Magne et al., 2002).

At present, all the commercial 3D printers for dental applications do not use FRC, but metallic materials, ceramics, and resins. Specifically, denture superstructures produced by additive manufacturing are mostly made of metallic materials. However, there are several reasons for using nonmetallic coronary restorations: metals expand and contract due to temperature changes in the mouth, have high thermal and electrical conductivity, high weight and density, high allergen potential, and shine to X‐ray.

The aim of this study was to evaluate the mechanical characteristics of FRC superstructure made by using a novel 3D printing technology able to create a reinforcing structure patient‐specific, more reliable, structurally optimized, and faster than conventional methods.

The process starts with a denture 3D model analysis that is inserted in an algorithm that generates a toolpath according to performance requirements and the geometries can be later fulfilled. The software simulates instructions to avoid collisions and sends data to a robot that with the attached tool head moves at the desired position while depositing a preimpregnated glass fiber composite. Once deposited on the build surface, an ultraviolet (UV) polymerization apparatus instantaneously activates the curing agent contained in the resin material. This fast polymerization allows the creation of complex geometry reinforcement placed along the arch following the contour and abutment geometries.

## 2 MATERIALS AND METHODS

To evaluate mechanical performances of 3D‐printed fiberglass a mechanical characterization of 3D‐printed material was performed.

**Figure 1:**
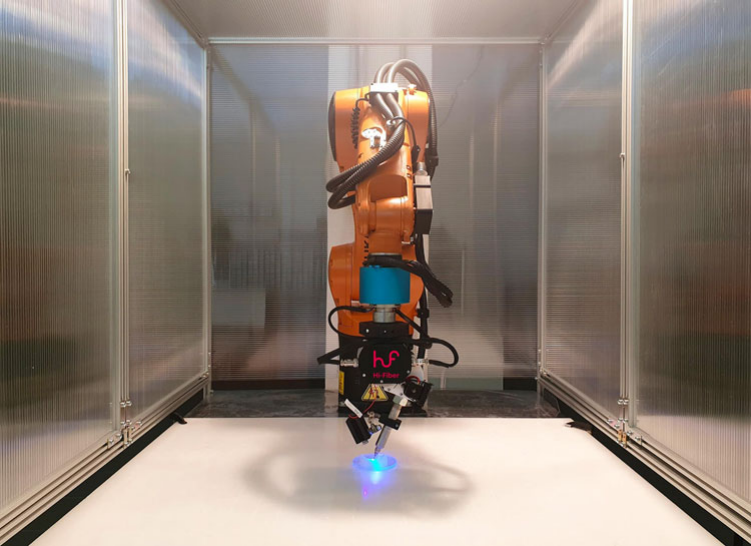
Three‐dimensional printing machine

Commercially available LuxaPrint Ortho Plus (supplied by DMG Chemisch‐Pharmazeutische Fabrik GmbH–a society that produce dental materials) resin was used as the matrix material. A couple of continuous glass fibers yarn 66 TEX S‐3 HPB (supplied by AGY; AGY is a leading global producer of fiberglass yarns and high‐strength fiberglass reinforcements used in a variety of composites applications), were used as reinforcement, and no additional surface treatments were done. The materials are already certified for dental application. A specific tool head and software were designed to 3D print with a robotic arm KUKA KR10 R900 sixx equipped with a KR C4 Compact controller (Figure 1).

### 2.1 Tensile test

We tested five specimens. A tensiometer (Zwick Z101, Ulm, Germany) was used to perform such tensile tests with a crosshead movement speed of 2 mm/s. The specimen configuration was rectangular with end tabs. The dimensions of the specimens are: *l*: 205 mm, *w*: 15 mm, *h*: 1 mm. The samples were 3D‐printed along the width direction using 2 perimeter of 0.5 mm each. The impregnated fibers bundles have a height of 0.295 mm and they are deposited in 51 layers to obtain a width of 15 mm. To prevent gripping damage tabs are applied to the samples. The specimens were clamped to the wedge grips of the testing machine and a tensile load was applied uniaxially to the specimen. Tensile test specimens have been produced in conformity with standard test methods for composite materials (Following ASTM D3039 standard). The continuous fibers were laid in the longitudinal direction of the specimen. The fibers set in this way can be considered an orthotropic material with the main directions of the fibers aligned with the main directions of the specimen.

### 2.2 Volumetric fiber content measurement

To have an estimation of the mechanical properties of the 3D‐printed specimens, the volumetric fiber content of the composite material was measured. Thermogravimetric analysis was performed on a sample of the 3D‐printed material and the result showed the weight percentage of the fibers after all the matrices had been burnt. The volumetric fiber content can be easily calculated from the weight percentage of the fibers as the specific weights of the fibers and the matrix are known. This procedure is performed on a sample and the mean volumetric fiber content of the 3D‐printed material is equal to 45.8%.

### 2.3 Denture reinforcement 3D printing process

**Figure 2:**
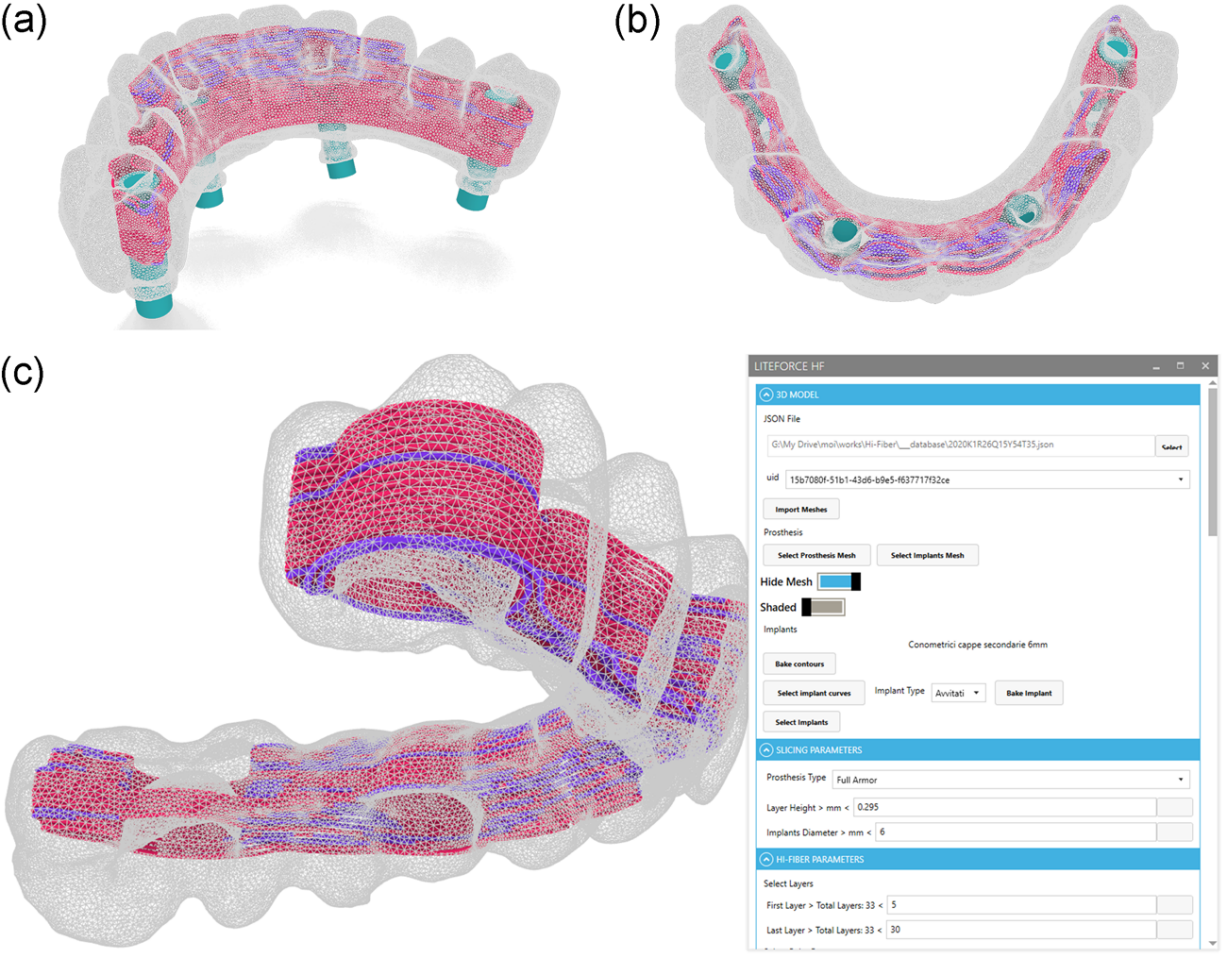
(a) The 3D model and path programmed, (b) highlight the near to implants path, (c) shows input parameters. 3D, three‐dimensional

Starting from the standard triangulation language file of the 3d scanned denture volume, the design of fiber reinforcement denture was obtained by using an algorithm. This algorithm is able to define a useful outline of the 3D scanned part and create a path offsetting the denture geometry and implants contours for all the layers that compose the 3d path (Figure 2).

**Figure 3:**
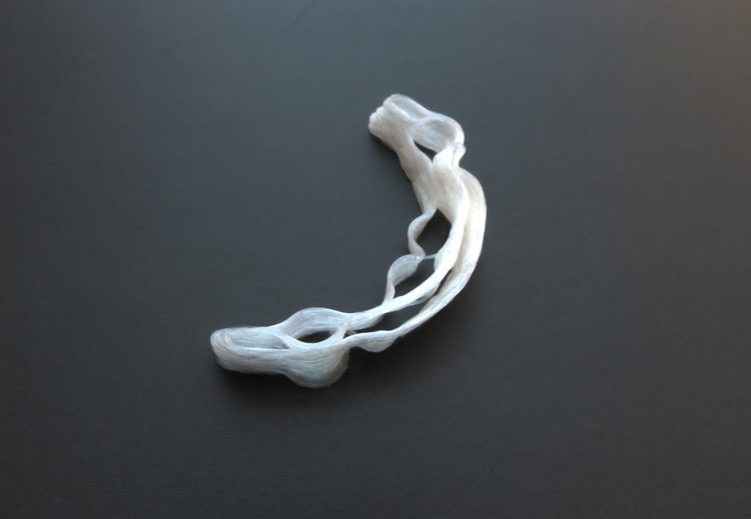
Three‐dimensional printed reinforcement structure

**Figure 4:**
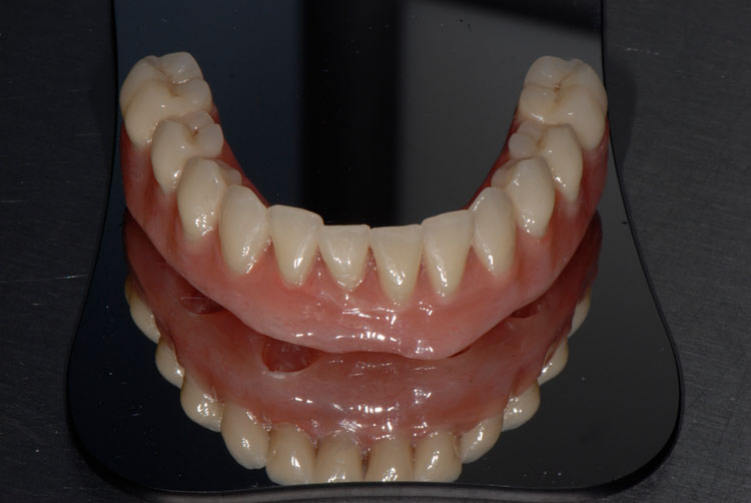
Three‐dimensional printed reinforcement structure finalized by Biart

The entire process is automatic and does not require human operation thanks to specific software programming. Initially, the robot prints layers of fiber which are immediately polymerized. This procedure takes about 30 min. Then the structure is placed in a UV oven for 3 min to complete the polymerization. Finally, it is washed with ethanol and ultrasounds to eliminate the uncured excesses. The obtained 3D‐printed reinforcement structure weight was about 2.4 g, and it was finalized by Biartlab (Figures 3 and 4).

**Figure 5:**
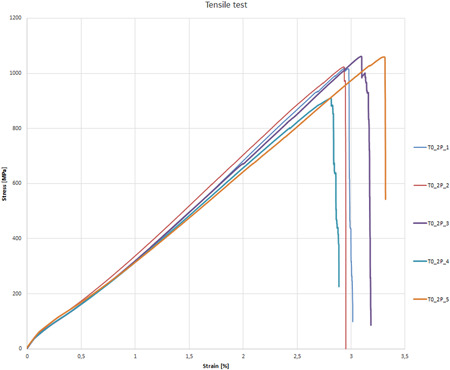
Specimens tensile test

## 3 RESULTS

### 3.1 Tensile tests

As shown in Figure 5, the stress–strain curve of the 3D‐printed continuous FRC specimens exhibit the typical behavior of unidirectional fibers composites. The failure is brittle and related to the rupture of a fiber bundle which causes the reduction of the cross‐section and the specimen failure. The tensile modulus, the tensile strength, and the tensile strain‐to‐failure were extrapolated from the experimental curves; the linear segment of the stress–strain curve was considered for the evaluation of the tensile modulus.

The mean values and standard deviation of the tensile modulus, tensile strength, and tensile strain‐to‐failure for the different specimens are shown in Table 1. The tensile modulus of the continuous fiberglass reinforced material was 33.4 ± 1.2 GPa. The specimens exhibited a tensile strength equal to 1014.9 ± 62.0 MPa and a tensile strain‐to‐failure of 3.1 ± 0.2%.

**Table 1:** The mean values and standard deviation of the tensile modulus, tensile strength, and tensile strain‐to‐failure for the different specimens

ID specimen	*S*_M_(MPa)	*E*_t_(GPa)	*e*_M_(%)
T0_2P_1	1020.4	34.5	3.0
T0_2P_2	1023.8	34.6	3.0
T0_2P_3	1061.3	32.1	3.2
T0_2P_4	909.4	32.1	2.9
T0_2P_5	1059.4	33.5	3.3
Average	1014.9	33.4	3.1
Standard deviation	62.0	1.2	0.2

### 3.2 Comparison with the mechanical theory

The mechanical behavior of a composite material can be estimated by means of the use of micromechanical models. The prediction of the longitudinal elastic modulus of an orthotropic continuous fibers composite material is done thanks to the parallel model. Knowing the fiber volume content (*V*_f_ = 45.6%) and the elastic modulus of both the fibers (99 GPa) and the matrix (no data available from the manufacturer; considering the minor influence of the exact data on the final result, it was assumed to be 2.5 GPa as an average elastic modulus of typical resin), the predicted elastic modulus is 46.5 GPa. By assuming that the tensile strength of the continuous composite in the longitudinal direction is given only by the reinforcing fibers, the Ultimate tensile stress can be estimated as *σU*_c_ = *σU*_f_ × *V*_f_. The ultimate tensile stress of the glass fiber is 3300 MPa. The predicted value of the ultimate tensile stress of the composite is about 1504.8 MPa.

Table 2 shows the comparison between the experimental values and the predicted ones. We found that the elastic modulus is 28.2% lower than what theory predicts, whereas the ultimate tensile stress value is 32.6% lower than the predicted value. In conclusion, the mechanical properties of the 3D‐printed CFRC are slightly lower than the theoretical values, but the results are in line with industrial‐grade materials for the specific application.

**Table 2:** The comparison between the experimental values and the predicted ones

	Theoretical value	Experimental value
Average elastic modulus (GPa)	46.5	33.4
Average ultimate tensile stress (MPa)	1504.8	1014.9

### 3.3 Denture reinforcement 3D printing process

The fiber structure obtained using the digital production process shows a great chemical adhesion with the resin used in the finalization, and an opaque layer was not required. Analyzing the final prosthesis from the esthetic point of view, fiber bundles allow great translucency of the entire system, very unique, especially in thin spaces. Furthermore, the fibers impregnated by resin show a behavior very similar to fluorescence because they can reflect UV rays. This reflection is not linear, due to the architecture of the fiber web and to the shape of a single fiber, the radiation is spread in a way very similar to what happens in the natural teeth.

## 4 DISCUSSION AND CONCLUSIONS

Fiberglass was introduced in the dental prosthesis a long time ago but its usage was not encouraged because it required a manual process, was time‐consuming, not repeatable, strictly dependent on the operator's abilities, and most importantly, uncertified.

By the present process of additive manufacturing, we have overcome these limitations and obtained a significant increase in the mechanical strength of the prosthesis, without increasing weight. The fiber paths are optimized as well as their density. Without interrupting the fibers the mechanical behavior is similar to what happens in the bridge steel cables, that sustain and transmit the forces all around the entire structure eventually dissipating before that forces pass through the pillars, in our case the implant connections. The prosthesis as we obtained after the esthetic envelope and teeth mount was completed and it is possible to imagine that it has a very new mechanical behavior with respect to the current solutions because of the chemical linkage between matrices material. We can assume that the entire prosthesis became a new composite structure, moreover, all the constituent materials act in the same way and cooperate with each other in responding to mechanical stress. In other words, all the prostheses became a bearing structure, including the teeth that are crossed by fibers. We can also assume that the novel reinforcement structure presented in this study will react as a shock absorber in the masticatory cycle, generating a better absorbance of loads if compared with a rigid metal or ceramic core prosthesis.

This particular shape and behavior are more typical of an endoskeleton instead of a more conventional prosthetic framework, for this reason, we prefer to call it *endostructure not mesostructure*.

Using a fiber bundle width of 0.5 mm and a layer height of 0.295, the final 3D‐printed reinforcement structure can be adopted also in complex cases in which the available space for reinforcement is limited, without compromising the mechanical resistance and aesthetical result.

Furthermore, a prosthesis obtained with this method is very easy to reline even intraorally. In conclusion, we can say that the outcomes we obtained endorse its performance both mechanical and esthetic.

## AUTHOR CONTRIBUTIONS

**Andrea Nicali**: Conceptualization; writing–original draft preparation. **Giulia Pradal**: Writing–review and editing, visualization. **Andrea Mantelli**: software. **Gianluca Brandolini**: investigation. **Marinella Levi**: supervision.

## CONFLICTS OF INTEREST

The authors declare no conflicts of interest.

## Data Availability

The datasets generated during and analyzed during the current study are available from the corresponding author on reasonable request.
